# Homeostasis and food craving in obesity: a functional MRI study

**DOI:** 10.1038/s41366-021-00920-4

**Published:** 2021-08-17

**Authors:** M. A. Stopyra, H.-C. Friederich, N. Lavandier, E. Mönning, M. Bendszus, W. Herzog, J. J. Simon

**Affiliations:** 1 Department of General Internal Medicine and Psychosomatics, Centre for Psychosocial Medicine, University Hospital Heidelberg, Heidelberg, Germany; 2 Department of Neuroradiology, University Hospital Heidelberg, Heidelberg, Germany

**Keywords:** Cognitive control, Obesity, Obesity

## Abstract

**Objectives:**

Food intake in obesity has been found to be reward-based and less contingent on homeostatic needs. Accordingly, previous studies investigating neural processing of food cues observed aberrant processing in reward- and control-related brain regions in obesity. To further investigate the relation between homeostasis and food intake, this study investigated the influence of glucose metabolism on the neuronal response during the regulation of food craving in participants with obesity.

**Methods:**

Twenty-five normal-weight and 25 women with obesity were examined on two occasions after receiving either water or glucose directly into the stomach using a nasogastric tube. Participants were blinded to the type of infusion and were required to refrain from eating for 16 h before each visit. An event-related fMRI paradigm was used to investigate the effect of intestinal glucose load on the neuronal response during the regulation of food craving.

**Results:**

A 2 × 2 mixed-model ANOVA revealed that craving regulation was associated with increased activation in fronto-parietal regions in participants with obesity when compared to healthy controls. However, this effect was observed independently from homeostatic satiety. A regression analysis revealed that the reduction of food craving was related to increased activation in the lingual gyrus in individuals with obesity following the infusion of water.

**Conclusions:**

In participants with obesity, the neuronal response during the regulation of food craving is associated with increased neural cognitive top-down control and increased visual food processing. Since this observation was independent from satiety status, our results indicate a reduced influence of homeostasis on neural processing during food craving in obesity. This study was registered on clinicaltrials.org: NCT03075371.

## Introduction

One of the major global health threats is the continuously increasing prevalence of overweight and obesity [1] with more than 1.9 billion adults suffering from overweight, of which 650 million are obese [2]. Given that mortality and morbidity steadily increase with surplus body weight [3]; understanding the neurophysiological mechanisms behind prolonged and excessive overeating is of fundamental importance to effectively prevent and treat adiposity. Previous research is indicative of alterations in neuronal reward processing, cognitive control as well as energy homeostasis as underlying factors of increased food intake in obesity [4].

In order to elucidate the neurobiological mechanisms associated with overeating, it is necessary to consider body weight regulation in its entirety as it involves the integration of homeostatic, reward-, and cognitive control-related processes. The etiology of obesity is related to abnormalities in peripheral metabolic signaling, which have been found to be both a consequence of excessive weight gain as well as predisposing factor [5]. For example, the “hunger hormone” ghrelin stimulates the initiation of eating, and baseline plasma ghrelin concentrations have been found to be decreased in individuals with obesity when compared to lean individuals [6]. In contrast to ghrelin, levels of circulating leptin are increased in individuals with obesity, who typically develop a resistance to leptin signaling [7]. Leptin-resistance develops as a consequence of a prolonged period of overeating [8], which may have damaging effects on the hypothalamus, a brain region crucial for the control of food intake [9]. Consequently, the hypothalamus becomes less sensitive to leptin, leading to sustained increase in leptin levels and increased food intake [10].

Despite the substantial impact of metabolic signals on eating behavior, food consumption is not solely driven by nutritional needs but also underlies volitional control as well as the impact of hedonic cues [11]. Neuroimaging studies found an exaggerated neural reactivity to high-caloric food cues in individuals with obesity [12, 13] and have demonstrated that appetitive food cues promote hedonic eating [14–16]. A hyper-responsive neural reward system may underlie the increased motivational importance of food stimuli, including brain areas such as the nucleus accumbens, striatum, amygdala, and the orbitofrontal cortex. Increased reactivity to food cues is further promoted by a hypo-activation of frontal regions, which are commonly implicated in response inhibition and cognitive control [4, 17]. Furthermore, the degree of brain activation in response to high-calorie food is positively related to subsequent weight gain [18, 19]. In particular, the dorsal striatum, a region commonly associated with reward anticipation and habit formation, shows increased activation in response to visual food cues in individuals with obesity [18]. In addition, dorsal striatal functional connectivity is increased in individuals with obesity and is positively related to food craving [20]. Given the heightened reactivity to food rewards in obesity, which has been found to be partially independent from physiological hunger [18], food cues can trigger stimulus-response-learned behavior driven by altered dopamine neuro-circuitry [21, 22]. These alterations are in turn associated with pathological overeating and obesity.

Taken together, investigating the interaction between homeostatic, hedonic as well as cognitive mechanisms is necessary to elucidate the pathophysiology of obesity. Previous functional magnetic resonance imaging (fMRI) studies have almost exclusively focused on neuronal responses to visual or taste food cues [23, 24], thereby disregarding the interactive effect of metabolic signals upon the neuronal response of cognitive control. However, cognitive and emotional factors are also involved in food choice and food intake. Especially sensory aspects of food ingestion such as sight or smell have been found to trigger learned habits or conditioned reward expectations [25], which in turn influence the valuation of and hedonic response to food [26]. Previous studies investigating the reaction to palatable food cues were unable to correct for these influences such as e.g., conditioned reward expectations, evaluation, learned habits, and situational contexts [4, 27]. Specifically, expectations about the consequences of eating (i.e., fullness, hedonic, or nutritive value) and memories of previous eating episodes, are both able to influence the hedonic response to particular foods [28]. Accordingly, the present study is specifically interested in the effect of metabolic status on the neuronal regulation of food craving by eliminating anticipatory effects of visual, olfactory, and oral cues. Given that obesity is associated with decreased sensitivity toward food reward [29] and metabolic signaling [30], we hypothesize that individuals with obesity display decreased responsivity in response to glucose administration in comparison to the normal-weight group. As food craving is a driving factor for overeating and the development of overweight and obesity [31], this study aims at further examining the neuronal processes underlying cue-induced food craving and the regulation of craving in participants with obesity. More specifically, we will examine the influence of intestinal glucose administration on the neuronal response to self-regulation of exteroceptive food cues in participants with obesity. This study can contribute to the understanding of the relationship between cognitive, homeostatic as well as hedonic signaling of food craving regulation in obesity.

## Methods

### Participants

Twenty-five female participants with obesity with a body mass index (BMI) of >30 and <40 kg/m^2^ and 25 age- and education-matched normal-weight (BMI > 19 and <25 kg/m²) participants underwent two functional MRI sessions. The age range for individuals with obesity was 19–45 years and in normal-weight individuals 20–47 years. Detailed demographic and clinical characteristics can be derived from Table 1. A total of four participants had to be excluded from fMRI analysis due to excessive head motion (>2 mm) (excluded number of healthy control participants (*N*_CON_) = 1, excluded number of participants with obesity (*N*_ADI_) = 3). All participants were screened by means of the Structured Clinical Interview for DSM-IV [32] for psychiatric disorders. All axes I and II disorders were excluded except of a history of major depressive disorder in participants with obesity (*N*_ADI_ = 3). At the time of the study, participants did not take any medication, which affected the central nervous system. Further exclusion criteria were left-handedness, contraindications for MRI, psychotropic medication, pregnancy, and male gender. The present study was approved by the ethics committee of the University of Heidelberg and it was in accordance with the ethical standards of the Declaration of Helsinki of 2008. Participants had to provide written consent in order to participate in the current study.

**Table 1:** Demographic and clinical characteristics of participants.

Variable	Normal-weight controls (*N* = 24)	Participants with obesity (*N* = 22)	*P*
Age in years, mean (SD)	24.99 (5.29)	27.09 (6.25)	0.224
BMI, mean (SD)	21.77 (1.48)	35.57 (3.88)	<0.001
Education in years, mean (SD)	12.87 (0.62)	12.32 (1.28)	0.064
BDI, mean (SD)	4 (3.83)	11.45 (8.43)	<0.001
EDEQ total score, mean (SD)	11.08 (9.29)	57.27 (22.24)	<0.001
EDEQ restraint, mean (SD)	2.29 (3.98)	10.54 (6.18)	<0.001
EDEQ eating concern, mean (SD)	0.5 (0.72)	5.95 (4.87)	<0.001
EDEQ weight concern, mean (SD)	2.54 (2.12)	14.27 (5.65)	<0.001
EDEQ shape concern, mean (SD)	5.75 (5.29)	26.5 (10.82)	<0.001

*BDI* Beck Depression Inventory, *BMI* body mass index, *EDEQ* Eating Disorder Examination Questionnaire.

### Questionnaires

At the beginning of the first fMRI session, participants were asked to fill out Beck’s Depression Inventory (BDI; [33]), as well as the German version of the Eating Disorder Examination Questionnaire (EDEQ; [34]). Before and after each fMRI session, participants had to indicate their degree of hunger on a 100 mm visual analog scale. After each fMRI session, participants were asked to guess which liquid (i.e., glucose or water) they were given.

### Procedure

The procedure, stimuli, and task have been reported previously [35–37]. At both study sessions, all participants had fasted for a minimum of 16 h, and fMRI scanning started around 12 p.m. On the first study session, all participants had to fill out questionnaires and undergo a clinical interview [32]. This was followed by the placement of a fine-bore nasogastric tube (Flocare Nutrisoft, Nutricia GmbH, Erlangen, Germany) placed with its tip in the gastric ventricle and fixed to the participants’ cheek. Each fMRI session started with a 5 min baseline scan, followed by the administration of 300 ml water or 75 g glucose dissolved in 300 ml of water (Accu-Chek^®^ Dextrose O.G.-T., Roche, Basel, Switzerland) through the nasogastric tube. The dose of glucose (75 g) was based on the Oral Glucose Tolerance Test, which is a standard method to assess glucose sensitivity, and previous studies have demonstrated that 75 g of glucose are sufficient to evoke neuronal responses [38–40]. The order of receiving glucose or water at the first fMRI session was counterbalanced and randomized across participants. Following liquid infusion, participants underwent 30 min of fMRI scanning to assess responsivity of the hypothalamus to glucose/water infusion (results are reported here; [35]). This was followed by the experimental task, which lasted ~17 min (results of healthy participants and patients with anorexia nervosa have been previously reported; [36, 37]). In order to ensure an appropriate physiological response to glucose, three blood samples were taken: 30 min prior to entering the scanner, 30 and 60 min after infusion of glucose or water.

### Biochemical analysis of glucose

Assessment of glucose concentrations was performed at the central laboratory of the University Clinic Heidelberg on a Siemens Advia 2400 device using the hexokinase method. Differences in blood glucose levels were assessed using a repeated-measures ANOVA with infusion type and time point as within, and group as between factors.

### Stimuli and task

At each fMRI session, participants were required to choose 8 out of 85 images, which depicted high-calorie food. More specifically, participants were asked to choose those images that they currently experienced craving for. Eight nonfood images were used as a control condition. The experimental paradigm was a modified version of an emotion regulation task [41]. The paradigm was implemented in an event-related design and consisted of two instructions. Participants were asked to either look attentively at the images (i.e., viewing condition) or downregulate their craving by means of distracting themselves with an arithmetic equation (i.e., distraction condition). After a fixation cross (jittered interval of 3000–4500 ms), the food or nonfood image was presented for 1000 ms. In the distraction condition, the induction was followed by the presentation of the arithmetic equation as a semitransparent overlay on the image for 6000 ms. Participants were instructed to solve the equation as fast as possible and indicate via a button press whether the equation (e.g., 4 + 8 − 2 = 11) was solved correctly or incorrectly. The viewing condition was initiated by viewing instructions (1000 ms), followed by the presentation of the image for 5000 ms. Each experimental condition was followed by a 9-point Likert scale craving rating for the previously depicted food image (or a desire rating in the case of nonfood objects) for another 4000 ms. The experimental paradigm consisted of 64 trials in total and each image was presented twice throughout the experiment.

### Functional MRI image acquisition

Functional MRI images were collected on a 3 Tesla Siemens Trio MRI scanner (Siemens Medical Solutions, Erlangen, Germany) at the University Hospital Heidelberg, Germany. Per study session, a total of 523 functional T2*-weighted images were acquired in an interleaved slide order with a voxel size of 3 × 3 × 4 mm, a repetition time (TR) = 2000 ms, echo time (TE) = 30 ms, and flip angle (FA) = 80°. Each volume consisted of 30 axial slices and a field of view (FOV) = 192 × 192 × 120 mm. Furthermore, we acquired a T1-weighted structural image with 192 slices, a TR = 1900, TE = 2.52 ms, and a FOV = 256 × 256 × 256 mm.

### Data analysis

Functional MRI data were analyzed with Statistical Parametric Mapping (SPM8, Wellcome Department of Imaging Neuroscience, London, United Kingdom) running on MATLAB Version 7.13 (Mathworks, Inc.). The pre-processing pipeline consisted of slice-time correction, realignment, spatial normalization to a standard stereotactic space and spatial smoothing with a full-width at half maximum Gaussian smoothing kernel of 8 mm. Structural images were segmented into gray matter, white matter, and cerebrospinal fluid partitions following co-registration with the mean T2* image and spatially normalized to the Montreal Neurological Institute standardized space.

At the single-subject level, the experimental conditions (i.e., food distraction, nonfood distraction, food viewing, nonfood viewing) were defined by stimulus onset and the duration of each experimental trial. Experimental conditions were modeled and convolved with the hemodynamic response function. Six movement parameters, the ratings, and induction phase were added as regressors of no interest. A high-pass filter with a cutoff of 128 s was used to remove low-frequency noise. The following contrasts of interest were calculated by means of a General Linear Model: (1) distraction from food—viewing food, (2) viewing food—viewing nonfood objects. In contrast to our previous investigations with the same group of healthy participants [36, 37], we controlled for distraction-specific brain activation by using the results from the contrast “distraction from nonfood objects—viewing nonfood objects” as an exclusive mask in all subsequent second-level analyses. Specifically, a binary mask was built based on the combined group results (thresholded at *P* < 0.05 cluster level family-wise error (FWE) corrected with a cluster-defining threshold of *P* < 0.001 uncorrected and minimal cluster size of *k* > 50) and a disjunction analysis (exclusive masking) was performed with this mask to exclude activation in brain regions observed during both the distraction from nonfood objects and distraction from food.

At the group-level analysis, a 2 × 2 mixed-model ANOVA (flexible factorial design) with group (participants with obesity, healthy controls) as a between-subjects factor and liquid type (glucose, water) as a within factor was calculated. Subject constants were modeled with a between factor “subject” to adjust for dependencies. Accordingly, we did not include nuisance variables in this model. Paired *t*-tests were used to examine the effect of glucose and water infusion independently in normal-weight controls and participants with obesity.

**Fig. 1:**
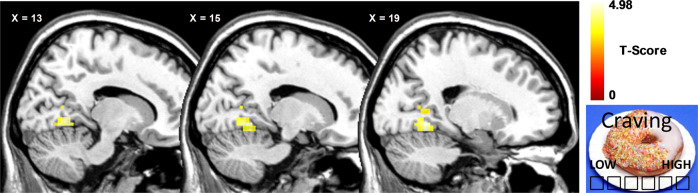
Whole-brain regression analysis with craving ratings in participants with obesity during distraction from food images compared to viewing of food images following water infusion. Craving ratings were positively correlated with lingual gyrus activation. Results significant at *P* < 0.05 cluster level family-wise error (FWE) corrected are reported, with a cluster-defining threshold of *P* < 0.001 uncorrected and minimal cluster size of *k* > 50.

Furthermore, we performed a whole-brain regression analysis for the contrast “distraction from food—viewing food” by modeling differences in craving ratings (craving ratings during distraction from food vs. craving ratings during viewing of food) as a covariate of interest. Depression scores were entered as covariates of no interest, regression analysis was calculated separately for both groups and for water and glucose infusion. For all fMRI analyses, results were inclusively masked with the mean gray matter image of all participants (mean binarized image calculated with ImCalc in SPM). Furthermore, only results significant at *P* < 0.05 cluster level FWE corrected are reported, with a cluster-defining threshold of *P* < 0.001 uncorrected and minimal cluster size of *k* > 50. Figure 1 was made using MRIcron software (http://people.cas.sc.edu/rorden/mricron/index.html).

Behavioral data were analyzed using SPSS (Version 25; SPSS Inc., Chicago, IL, USA). Group differences in psychometric scales and demographic variables were assessed using independent two-sample *t*-tests. Differences between groups in pre–post hunger ratings were assessed using a repeated-measures ANOVA, with group as a between and infusion type (glucose vs. water infusion) as well as time point (before vs. after scanning) as within factors. Craving ratings during the task were assessed using a repeated-measures ANOVA with infusion type as a within and group as a between factor.

## Results

### Behavioral results

We observed no differences between groups regarding age and education years, but a higher BMI as well as BDI and EDEQ scores in participants with obesity (see Table 1). Participants remained unaware of the type of liquid administered during each session, since both groups guessed at chance level at the first (normal-weight controls: *χ*^2^ = 0.043, *P* = 0.835, participants with obesity: *χ*^2^ = 0.220, *P* = 0.639) and second visit (normal-weight controls: *χ*^2^ = 2.291, *P* = 0.13, participants with obesity: *χ*^2^ = 0.188, *P* = 0.665).

### Hunger ratings

We failed to observe a significant interaction effect in our repeated-measures ANOVA (group × infusion type × time point, *P* = 0.779). Furthermore, we did not observe a significant change in hunger ratings pre–post scanning during water or glucose infusion in the whole sample (*Ps* > 0.062). When assessed separately, only participants with obesity displayed a significant increase in hunger ratings following water infusion (*t*(21) = 2.54, *P* = 0.019, see Table 2).

**Table 2:** Descriptive statistics for hunger/craving ratings and blood glucose levels.

Variable	Normal-weight controls (*N* = 24)	Participants with obesity (*N* = 22)	*P*
Hunger before water infusion	50.78 (26.05)	51.14 (22.31)	0.961
Hunger after water infusion	52.58 (28.15)	61.61 (20.89)	0.230
Hunger before glucose infusion	56.58 (23.42)	51.89 (25.35)	0.521
Hunger after glucose infusion	51.00 (26.11)	52.42 (28.68)	0.863
Craving during food distraction—water	7.27 (0.74)	6.93 (1.06)	0.220
Craving during food viewing—water	7.35 (0.68)	7.22 (0.98)	0.598
Craving during food distraction—glucose	7.01 (0.97)	6.59 (1.59)	0.289
Craving during food viewing—glucose	7.04 (0.94)	6.95 (1.45)	0.814
Blood glucose prior water infusion	83.73 (6.22)	84.45 (7.75)	0.733
Blood glucose 30 min post water infusion	82.45 (5.29)	81.40 (6.19)	0.559
Blood glucose 60 min post water infusion	82.82 (5.94)	81.95 (6.31)	0.646
Blood glucose prior glucose infusion	84.09 (4.61)	84.59 (7.57)	0.793
Blood glucose 30 min post glucose infusion	146.50 (19.48)	132.52 (26.35)	0.056
Blood glucose 60 min post glucose infusion	138.80 (29.34)	133.77 (37.42)	0.629

### Craving ratings

There was no interaction between group, infusion type, and experimental condition (viewing *vs*. distraction) during craving ratings of food pictures (*F*(1,44) = 0.076, *P* = 0.785). Furthermore, there was no effect of group on infusion type and experimental condition during craving rating (*Ps* > 0.067). There was no main effect of infusion type on craving ratings in the whole sample (*F*(1,44) = 1.66, *P* = 0.204), but a main effect of experimental condition on craving ratings (*F*(1,43) = 7.43, *P* = 0.009), with reduced craving rating during distraction when compared to viewing in the combined sample (*t*(91) = −2.57, *P* = 0.012, see Table 2).

### Blood glucose analyses

Due to technical difficulties, blood glucose levels could not be determined for all three measurements points in some participants. Details about missing values are given along the respective test results. We observed a significant interaction between infusion type (glucose vs. water infusion) and time point (before, during and after scanning; *F*(2,78) = 104.9, *P* < 0.001, missing values in healthy controls: 2, missing values in participants with obesity: 3), but no significant effect of group on the infusion type × time point interaction (*P* = 0.291). Both groups showed a significant increase in blood glucose values following glucose infusion (blood glucose after scanning compared to baseline, normal-weight controls: *t*(21) = 8.55, *P* < 0.001, missing values: 2 participants with obesity: *t*(21) = 4.3, *P* < 0.001, missing values: 0). Both groups did not differ in baseline blood glucose levels during both fMRI measurements (*Ps* > 0.613, missing values in both groups: 0). Furthermore, both groups displayed baseline blood glucose levels typically observed in a fasted state in healthy individuals [42]. For detailed descriptive statistics see Table 2.

### fMRI results

#### Group differences

We observed no significant interaction between group and liquid type for both contrasts of interest. However, for the contrast distraction from food compared to viewing food, we observed a significant effect of group in the right dorsolateral prefrontal cortex (DLPFC), medial occipital cortex, bilateral inferior parietal lobule, anterior prefrontal cortex, and middle frontal gyrus (see Table 3). A post hoc test comparing both groups revealed that activation in these regions was increased in participants with obesity. However, it has to be noted that this analysis was artificially overpowered since we included two contrast images for each participant (for both the water and glucose condition). For the contrast viewing food compared to viewing nonfood objects, we observed a significant effect of group in the occipital cortex, which was stronger in normal-weight controls (see Table 3).

**Table 3:** Repeated-measures mixed-model ANOVA results; significant effect of “group” on BOLD response during distraction from food and viewing of food.

Contrast/brain regions	*z* value	*P* value	*k*	*x*	y	*z*
Distraction from food compared to viewing food						
Dorsolateral prefrontal cortex	5.91	<0.001	184	45	41	26
Medial occipital cortex	5.41	0.001	181	−6	−85	6
Right inferior parietal lobule	5.39	0.001	323	39	−43	34
Left inferior parietal lobule	5.15	0.003	229	−48	−46	58
Anterior prefrontal cortex	5.08	0.004	95	−3	62	14
Middle frontal gyrus	4.88	0.009	81	36	8	62
Viewing food compared to viewing nonfood objects						
Medial occipital cortex	5.17	0.002	149	−15	−85	−2

*k* = cluster size (voxels). Results significant at *P*_FWE_ < 0.05 are reported, with a cluster-defining threshold of *P* < 0.001 uncorrected and minimal cluster size of *k* > 50.

Importantly, these effects were observed independently from the type of infusion since we found no interaction between group and liquid type. Accordingly, post hoc tests comparing groups during glucose and water infusion revealed no significant results.

#### Within group results

We observed no differences between glucose and water infusion in participants with obesity for both contrasts of interest. Whilst we observed no significant differences between glucose and water infusion in normal-weight controls during the contrast viewing food compared to viewing nonfood objects, we observed increased activation in the brain stem (*t* = 5.04, *k* = 87, *P*_FWE_ = 0.041, *x* = −9, *y* = −34, *z* = −22) and bilateral nucleus caudatus (*t* = 4.74, *k* = 93, *P*_FWE_ = 0.033, *x* = 9, *y* = 17, *z* = −2) following glucose infusion when compared to water infusion for the contrast distraction from food compared to viewing food.

#### Regression analysis

We observed no relation between brain activation during distraction from food compared to viewing food and craving ratings in healthy controls following both water and glucose infusion. Participants with obesity showed no relation between craving ratings and brain activation following glucose infusion; however, following water infusion, we observed a positive relation between differences in craving ratings (i.e., craving during viewing of food vs. craving following distraction from food) and activation in the lingual gyrus (*t* = 4.98, *k* = 96, *P*_FWE_ = 0.024, *x* = 24, *y* = −52, *z* = 2).

## Discussion

The present study examined the effect of intestinal glucose administration on the neuronal regulation of cue-induced food craving while bypassing eating-related cognitions in women with obesity. We did not observe differences in the neuronal regulation of food craving between glucose and water infusion in women with obesity. However, we observed a positive association between food craving reduction and activation in the lingual gyrus during the distraction from food images following water infusion.

We observed neuronal differences in the normal-weight group between the infusion of glucose and water during the distraction from food. More specifically, glucose administration increased activation in the brain stem and the bilateral nucleus caudatus. While the brain stem receives projections from the hypothalamus and therefore, highly contributes to multiple facets of energy homeostasis [43, 44], the caudate nucleus is involved in the expectation of a reward [45, 46]. Accordingly, our findings indicate a satiety-dependent interaction between glucose signaling and hypothalamic functioning as well as an adequate signaling from the hypothalamus to the rest of the brain. In contrast to normal-weight women, the neuronal regulation of food craving was independent of metabolic status in participants with obesity. This result is in line with a previous investigation of our research group, where we investigated the same participants and found that obesity is related to a blunted hypothalamic reactivity in response to glucose infusion [35], which points to a decreased or desensitized neuronal reactivity to glucose metabolism. The present study extends these findings to neuronal top-down control involved in food craving. In fact, previous studies have demonstrated a blunted neuronal response to visual food cues as well as a negative association between BMI and brain activation upon the receipt of a milkshake [18, 47]. Moreover, a reduced influence of satiety in obesity has also been confirmed by endocrinological studies [48]. Participants with obesity compared to normal-weight participants do not exhibit the expected decrease in leptin and ghrelin secretion following food consumption [49, 50]. Similarly, our findings may allude toward an impaired neuronal suppression of appetite following glucose infusion, which in turn reinforces increased food intake and weight gain. The neuronal desensitization to metabolic changes in blood glucose levels are most likely mediated by neuro-endocrinological signals such as ghrelin and leptin. Therefore, impairment in central satiety signaling might play a role in dysfunctional craving regulation in obesity.

In line with our expectations, glucose infusion did not affect subjective satiety ratings. This observation may be caused by the absence of cognitive processing and sensory signaling, which have previously been associated with weaker satiety responses [51]. We cannot exclude that a higher dose of glucose might have evoked significant effects on subjective satiety. However, the present study aimed to investigate the effect of homeostatic satiety independent of subjective satiety on neuronal response. Our findings underline the importance of the cephalic phase of gastric secretion and nutritional value as essential contributory factors to the subjective experience of satiety and associated neuronal responses. In fact, Crézé and colleagues argue that congruent taste signaling and caloric value are necessary to evoke a physiological as well as behavioral satiety response [52].

Both groups showed a similar reduction of food craving following distraction. Although we observed no group differences in neural processing during distraction when comparing water and glucose infusion separately, when pooling the data across conditions (i.e., water and glucose), the distraction from food craving for appetizing food images was associated with increased activation in fronto-parietal regions (including the DLPFC) related to self-regulation in individuals with obesity. Previous studies found that the DLPFC constitutes a core brain region in dietary self-control and is involved in the downregulation of appetitive incentives [53–55]. Therefore, our results might indicate that individuals with obesity require additional cognitive resources to achieve a similar degree of craving reduction as normal-weight participants. However, given that group differences were only present when pooling across conditions, these findings should be considered with caution as pooling between conditions artificially increases statistical power.

Moreover, increased activation in the lingual gyrus was related to a stronger reduction of food craving in women with obesity. The lingual gyrus is mainly involved in attentional processing but has also been observed during visual processing of food cues when compared to nonfood stimuli [56, 57]. Furthermore, an investigation by Aviram-Friedman and colleagues [58] found that participants with binge eating disorder display increased activation in the lingual gyrus during viewing of both low- and high-caloric food images. The authors discussed the involvement of visual regions as an indication of involuntary attention to food. In addition, the lingual gyrus is activated by visual craving-inducing cues such as alcohol, cigarettes, drugs, gaming, and food [59–61]. Although speculative, an increased allocation of attentional resources during food craving regulation might indicate the need for increased cognitive processing during craving reduction in obesity. However, since we observed no differences between our groups in craving reduction, it remains uncertain whether the association between lingual gyrus activation and craving reduction in participants with obesity is indicative of an additional requirement of neuronal resources to reduce craving.

This study exhibits several limitations. The present study did not assess executive functioning and possible differences between individuals with obesity and normal weight in executive functioning might have affected our neuroimaging findings. Moreover, differences in depressive symptoms between both groups might have confounded the fMRI results, as depression can affect the processing of rewarding stimuli [62]. Furthermore, we only examined women and since sex-related differences in neuronal food processing have previously been observed [63], our results should be generalized with caution. Another limitation might be the circumstance that we did not control for the menstrual cycle, despite indications that changes in estradiol and progesterone influence neural food processing [64, 65]. In addition, we did not collect data on energy need and expenditure and cannot rule out that this might have affected glucose metabolism. Furthermore, we cannot rule out the influence of habituation effects on our results, since participants were required to choose only eight food images. The present study only investigated one single food craving regulation strategy. However, there appear to be inter-individual preferences with regards to different strategies [66, 67]. Last, it should be mentioned that participants might have also (explicitly or implicitly) regulated their food cravings during the viewing condition, since individuals with obesity often exhibit a history of dieting experiences, which may impact cognitive evaluation of food pictures [68, 69]. Therefore, future studies should examine the impact of previous dieting experiences on regulation strategies during food craving.

## Conclusion

Taken together, our data support the conclusion that in individuals with obesity, neuronal processing during food craving regulation is independent of physiological satiety. These findings add to the observation of a central neuronal resistance to glucose signaling in obesity and underline the importance of homeostatic alterations for the maintenance of obesity. Our results furthermore suggest increased neuronal top-down control as well as visual attentional processing during craving reduction in obesity. Additional studies are needed to advance our insight of the relationship between food craving, craving regulation, and homeostatic signaling. Understanding the association between eating behavior and neuronal activation is of upmost importance in order to foster novel treatments for obesity and overweight.
